# Lifetime Prevalence of Psychiatric Disorders among Parents of Children with Bipolar I Disorder: Parental Difference

**DOI:** 10.1155/2014/256584

**Published:** 2014-11-09

**Authors:** Shahrokh Amiri, Mohammad Ali Ghoreishizadeh, Yasaman Alavizadeh, Farnaz Saedi

**Affiliations:** ^1^Clinical Psychiatry Research Center, Tabriz University of Medical Sciences, Tabriz, Iran; ^2^Department of Psychiatry, Razi University Hospital, Elgoli Road, Tabriz 51677, Iran; ^3^Tabriz University of Medical Sciences, Tabriz 51677, Iran

## Abstract

*Background*. Evaluation of family system is an important area in the context of child and adolescent mental health. This study aimed to estimate psychiatric disorders in parents of children and adolescents with bipolar I disorder (BID). *Methods and Materials*. In this cross-sectional study, during 2012-2013, all of the children and adolescents diagnosed with BID based on Kiddie Schedule for Affective Disorders and Schizophrenia-Present and Lifetime Version were included. All of the parents (both mother and father) were evaluated by Structured Clinical Interview for *DSM-IV-TR*. *Statistical Analysis*. Prevalence rates are reported and independent-sample *t*-test and chi-square test were used when appropriate. *Results*. A total of 108 families were interviewed. 25% of mothers and 33% of fathers met the criteria for at least one psychiatric disorder, with major depressive disorder, BMD, and cluster B personality disorder being more prevalent. Fathers were more likely to receive a dual psychiatric diagnosis. Cluster B personality disorder and substance dependence were more prevalent among fathers while major depressive disorder was more prevalent among mothers. *Conclusion*. This study confirmed a higher prevalence of psychiatric disorders in parents of children with BID and emphasizes parental evolution.

## 1. Introduction

Childhood is a fundamental phase of life assembling the future personality. However it could be accompanied by psychiatric problems influencing not only the development of child, but their families as well. Bipolar I mood disorder (BID) is one of the psychiatric disorders that may begin during childhood and is considered as a chronic and severe psychiatric disorder. Recent studies indicate a rapid increase in the diagnosis of bipolar disorders in youth [1].

Psychiatric disorders in families follow a multidirectional plan and the importance of the entire family system in the context of child and adolescent mental health is well recognized. Psychiatric conditions of parents may strongly influence the behavior of their children [2] and studies emphasize the importance of screening parents when their children receive a psychiatric evaluation [3]. Genetic factors play an important role in pathogenesis of psychiatric disorders as well and are shared between parents and children. Thus offspring of parents with different kind of psychiatric disorders are at higher risk for further psychiatric problems [4, 5].

Several lines of evidence support this connection for bipolar mood disorder [6]. Most types of parents' self-reported psychopathology symptoms are reported to have a role in the prognosis of behavioral, social, and emotional outcomes of preschoolers with behavior problems [7]. Parental depression is associated with early onset irritability, predicting later anxiety and depression in offspring [8]. A recent study confirmed that family characteristics increase the risk of bipolar disorder in children, with paternal substance use problems, with maternal depression, and through poor relationship with parents and conflict within the family being the most important factors [9]. The risk of suicide attempt in children is reported to carry direct clinical importance in case of exposure to parental psychopathology and suicidal behavior [10].

All of these studies suggest that higher psychopathology is expected in parents of children with mood disorder, while mothers' and fathers' problems may have different values [11]. Despite these lines of evidence, very few studies focus on parental psychopathology of children with bipolar mood disorders. This point of view will reveal the most important disorders to be focused on both parents. Detection of such psychopathology will be of assistance in the treatment of offspring as well. Thus the current study aimed to evaluate both parents of children with established bipolar I disorder, in regard to lifetime psychopathology as well as probing any difference between mothers and fathers.

## 2. Materials and Methods

This was a cross-sectional study of children and adolescents who were admitted to child and adolescent psychiatric ward of Razi Hospital, Tabriz, Iran, during 2012-2013. Razi Hospital is a tertiary referral (Tabriz University of Medical Sciences) and the only mental hospital in northwest of Iran with a ward dedicated to children.

The proposed study was approved by the regional ethical committee. A written consent was obtained from parents of participated children after comprehensive explanation about the aim of the study.

### 2.1. Participants

All of the children and adolescents (aged under 18) with a diagnosis of bipolar I disorder were enrolled. Patients with intellectual disability (IQ < 70) and any other psychiatric disorder (except for attention deficit/hyperactivity disorder and oppositional defiant disorder as well-known comorbid conditions for BID) were excluded. History of a serious head injury or any sensory deficit and nonattendance of one parent for evaluation also resulted in exclusion. After establishment of the diagnosis in patients, all of the parents (both mother and father) were invited to take part in the study.

The sample size was estimated to be 107 pair of parents according to Power and Sample Size program considering *α* = 0.05, *P* = 0.80, *P*_0_ = 0.6, and *P*_1_ = 0.4.

### 2.2. Measures

All of the diagnoses were made using the* DSM-IV-TR* criteria. Along with a thorough physical examination, developmental milestones, evaluation of blood chemicals, toxicology, neuroimaging, or electroencephalography (if clinically suspected), a semistructural psychiatric interview was carried out by a child and adolescent psychiatrist using the Farsi (Persian) version of Kiddie Schedule for Affective Disorders and Schizophrenia-Present and Lifetime Version (*K-SADS-PL*) [12] for diagnosing bipolar I disorder (BID) or any psychiatric comorbidities.

All of the parents were evaluated for any psychiatric disorder (on axis I or axis II) by the means of Structured Clinical Interview for* DSM-IV-TR* (*SCID-IV*). The psychometric properties of the Farsi (Persian) version of this questionnaire are described elsewhere [13].

### 2.3. Statistical Analysis

The prevalence of psychiatric disorders in mothers and fathers is reported and their characteristics are compared. SPSS Version 16 was used for the analysis. Data are given in mean (standard deviation) or number (percentage) where appropriate. Independent-sample* t*-test and chi-square test were used when appropriate. For all statistical tests, differences and correlations were considered to be statistically significant at *P* < 0.05.

## 3. Results

### 3.1. Children and Adolescents

A total of 108 children and adolescents with BID and their parents were eligible for this study. Children and adolescents included 57 boys (52.8%) and 51 girls (47.2%). Their mean age (SD) was 13.04 (2.85) years ranging from 7 to 18. All of them were students and educational breakdown was reported for 58.3% of these children.

### 3.2. Parental Differences

Mean age (SD) of fathers was 46.4 (6.4) that was significantly higher than mean age (SD) of mothers [42.3 (5.6), *P* < 0.001]. Level of education was not notably different between mothers and fathers (*P* = 0.21): 8 (7.4%) fathers and 17 (15.7%) mothers were uneducated, 92 (85.2%) fathers and 86 (79.6%) mothers were undergraduate, and 8 (7.4%) fathers and 5 (4.6%) mothers were postgraduate. More than 77% of families were resident in rural area.

During the psychiatric evaluations, fathers were more likely to receive a dual psychiatric diagnosis (*P* = 0.03, *χ* ^2^ = 5.14). One psychiatric disorder was diagnosed in 30 (27.8%) fathers and 28 (25.9%) mothers while 6 (5.5%) fathers (but none of mothers) fulfilled the criteria for two psychiatric diagnoses. However mothers and fathers did not have a difference regarding previous admission to a mental hospital. History of one psychiatric admission was reported by 14 fathers and 9 mothers, and 3 fathers and 4 mothers had been admitted twice (*P* = 0.66).

Results of psychiatric evaluation of parents are described in Table 1, describing lifetime prevalence (including present diagnoses). Cluster B personality disorder, any personality disorder, and any substance dependence were more prevalent among fathers compared to mothers. Major depressive disorder (MDD) was more prevalent among mothers.

A logistic regression model including age (*P* = 0.531), gender (0.144), educational level (0.287), and place of residence (rural versus urban, *P* = 0.570) showed that none of these had an independent effect on receiving a lifetime psychiatric diagnosis in this study sample.

## 4. Discussion

This study evaluated parents of children with BID and revealed that 25% of mothers and 33% of fathers met the criteria for at least one psychiatric disorder, with MDD, BMD, and cluster B personality disorder being more prevalent.

The main focus of this study was the difference between mothers and fathers of children with BID. Few studies have appraised this issue and methodological differences exist across studies (e.g., method of assessing and classification of psychopathology in parents and children, type of sample recruited). Results of several studies indicate stronger associations between maternal than paternal psychopathology and the presence of internalizing (but not externalizing) problems in children [3, 14]. Maternal symptoms [3] and maternal depression in particular [11] are frequently reported to be associated with child psychopathology in general. Our results confirmed this relationship as MDD was significantly more prevalent among mothers. This result may be influenced by our exclusion criteria which excluded widowed or divorced mothers (father not attended). Such difference was not observed for BMD.

A meta-analysis addressed researches on parental depression and emphasized the importance of paternal depression in relation to offspring internalizing and externalizing psychopathology [15]. The prevalence of MDD in fathers was lower than in mothers in our study but is still higher than general population of Iran, as described later.

Substance problems were more common among fathers of children with BID. According to previous studies, the independent factor influencing children of substance dependent fathers is the family problems caused by this situation [16]. Additionally, it is not surprising that cluster B personality disorder was also more prevalent among fathers. These results are also compatible with previous studies reporting that depressed mothers with depression tended to partner with antisocial fathers [17]. This combination (depression in mothers and antisocial behavior in fathers) needs to be a focus of intervention as both are significantly and independently associated with offspring depression and conduct disorder [17].

In comparison with results of an epidemiologic survey of psychiatric disorders in Iran [18], the prevalence of mood disorder was higher in parents of children with BID as well as psychotic disorder in mothers, but anxiety disorder in both parents and psychotic disorder in fathers were less prevalent. In the mentioned study which evaluated 25180 individuals based on* DSM-IV*, the prevalence of any mood disorder (both bipolar and unipolar) is reported to be 2.01% in men and 4.95% in women, any psychotic disorder is 0.67% in men and 1.12% in women, and anxiety disorder is 4.15% in men and 12.48% in women. The overall prevalence of any psychiatric disorder was reported to be 10.8% in this survey which is also lower than our study sample. There are no population based reports about prevalence of personality disorders in Iran.

A higher rate of psychiatric disorder in fathers (compared to mothers) is noted in our study which seems different from the described survey [18]. However it might be a result of different categorization, as personality disorders and substance related disorders are not reported in that survey where both were dissimilar between mothers and fathers.

Similar reports about parental psychopathology in children with different kinds of psychiatric disorders are available. Their results are disorder specific but have also some similarities. For example, parents were significantly more likely to have ADHD, Tic disorder as well as antisocial personality disorder, and problems with alcohol and drug abuse [19, 20]. Parental psychopathology is also described when subsyndromal problems are present in children. Low positive-emotionality in children was associated with maternal mood disorders [21]. In addition to the information that these studies provide about family genetic risk factors for distinct disorders, their common conclusion is that a comprehensive service is needed to address both psychiatric condition of affected children and the mental health needs of their parents.

The main limitation of this study was clinical based nature of patient recruitment. Most of children and adolescents with BID from northwest region of Iran are admitted to our center; however, this population is not necessarily all of these patients, especially patients with milder symptoms and from a low social level with little information about mental disorders. We also included families with married parents and pairs that were both available for a clinical interview. Thus, behavioral problems in parents may be underestimated in our study, as fathers or mothers with serious mental disorders may already be missed. On the other hand, structured psychiatric interview with both parent and child remains as an advantage for this study.

In conclusion, this study evaluated parents of children and adolescents with BID and found that 25% of mothers and 33% of fathers met the criteria for at least one psychiatric disorder. MDD, BMD, and cluster B personality disorder were the most prevalent disorders. Mothers were prone to MDD and substance use problems and cluster B personality disorder was more prevalent among fathers. These results suggest psychiatric evaluation of parents with a child with BID as well as primary prevention for children of parents with aforementioned disorders.

## Figures and Tables

**Table 1:** Prevalence of psychiatric disorders among parents of a child with BID and the comparison between mothers and fathers.

	Father	Mother	*χ* ^2^	*P*	Any parent
Major depressive disorder	3 (2.8)	11 (10.2)	4.88	**0.02**	14 (6.5)
Dysthymic disorder	0	1 (0.9)	1.005	1.00	1. (0.5)
Postpartum depression	—	2 (1.9)	—	—	—
Bipolar I disorder	8 (7.4)	6 (5.6)	0.30	0.58	14 (6.5)
***Mood disorders***	11 (10.2)	20 (18.5)	3.05	0.08	31 (14.4)
Schizophrenia	3 (2.8)	0	3.04	0.24	3 (1.4)
Schizoaffective	1 (0.9)	1 (0.9)	0	1.00	2 (0.9)
***Psychotic disorders***	4 (3.7)	1 (0.9)	1.84	0.36	5 (2.3)
Adjustment disorder	0	0	—	—	0
Obsessive-compulsive disorder	2 (1.9)	2 (1.9)	0	1.00	4 (1.9)
Posttraumatic stress disorder	1 (0.9)	0	1.00	1.00	1 (0.5)
Anxiety disorder-NOS	0	1 (0.9)	1.00	1.00	1 (0.5)
***Anxiety disorders***	3 (2.8)	3 (2.8)	0	1.00	6 (2.8)
***Substance dependence***	6 (5.6)	0	6.17	**0.02**	6 (2.8)
Cluster A personality disorder	1 (0.9)	0	1.00	1.00	1 (0.5)
Cluster B personality disorder	12 (11.1)	3 (2.8)	5.80	**0.02**	15 (6.9)
***Personality disorders***	13 (12.0)	3 (2.8)	6.75	**0.01**	16 (7.4)
Intellectual disability	3 (2.8)	1 (0.9)	1.01	0.62	4 (1.9)
